# The ethnobotanical domain of the Swat Valley, Pakistan

**DOI:** 10.1186/s13002-018-0237-4

**Published:** 2018-06-08

**Authors:** Kishwar Ali, Nasrullah Khan, Inayat-Ur Rahman, Waqar Khan, Murad Ali, Nisar Uddin, Mohammad Nisar

**Affiliations:** 1 Department of Botany, University of Malakand, Chakdara Dir Lower KP, Pakistan; 2 Department of Botany, Hazara University Mansehra, Mansehra, Pakistan

**Keywords:** Ethnobotanical domain, Swat Valley, Statistical indices, Khyber Pakhtunkhwa, Pakistan

## Abstract

**Background:**

This study contributes to the current ethnomedicinal knowledge of the Swat Valley, Pakistan. District Swat possesses remarkable biodiversity owing to its varied topographical and climatic conditions, prompting a distinct human-plant association. Our hypothesis is that the presence of such a great biodiversity has shaped into a formal ethnobotanical culture in the area transmitted through generations. We suspect that the versatility of some plant species has greater influence on the culture. Therefore, the prime objective of the study is to understand this unique human-plant relationship in the valley and to create scientific roots for the selection and practice of herbs in the ethnobotanical domain of the district.

**Methods:**

Primary data were collected using questionnaires and face-to-face interviews with the locals. The data collected were used for calculating some important indices, i.e. relative frequency of citation (RFC), participant agreement ratio (PAR), frequency of citation (FC), Smith’s Salience Index (SI), Relative Importance Index (RII), Cultural Value Index (CVI) and a newly proposed, Ali’s Conservation Priority Index (CPI). Index scores were used as key identifier of the ethnobotanically important plants of the area.

**Results:**

Residents of the Swat Valley have listed plant uses in 15 use categories. Around 9% of the respondents have a common consensus on the selection and use of plants for the treatment of evil eye with similar results for body cuts (8.2%) followed by psychological/neural ailments (8.0%). Respondents agree that *Berberis lyceum* Royle. dominates in all five indices. *Skimmia laureola* Franch. also constitutes one of the central plants of the ethnobotanical domain, ranking second in the SI, fifth in the RII, seventh in CVI, and third in the Cultural Importance Index. It holds the thirty-fifth position in the CPI. Over 80% of the population treat different diseases with herbal remedies. In the common ethnobotanical domain of the area, plants like *Mentha longifolia* L., *Berberis lyceum*, and *Skimmia laureola* are very important and have high salience and importance values, thus suggesting these plants are versatile for their uses in the study area.

**Conclusion:**

In conclusion, only some plant species are prioritised for their use in the ethnobotanical domain of the community. Medicinal and aromatic plant (MAP) usage is widespread in the Swat Valley. The ethnobotanical knowledge could be used as a tool to understand the adaptability of a specific taxon in the area and the possible conservation risk to their existence.

## Background

Plants and humans have a close and inseparable connection, not just ecologically, but socio-culturally since the existence of human beings. Plants in general and high-value medicinal and aromatic plants (MAPs), specifically, have a long history of use as a source of cheap and effective remedy for various ailments [1]. The use of MAPs can be traced back 7000 years [2]. Plants have always been an integral part of various cultures, i.e. the Sumerian clay tablet, a 4000-year-old medical script, documents the most primitive known plant therapies for various ailments.

Like other parts of the developing world, a significant portion of the population in Pakistan use medicinal plants traditionally for treating ailments and health disorders. There is an immense volume of literature available where a variety of studies have been carried out to document the intricate relationship between MAPs’ use in the aboriginals’ sociocultural and religious practices in various parts of Pakistan. Some of these findings suggest that the use of herbal therapies is directly or indirectly linked to the culture of the area [3–5], but no one has presented any sound proof of this relationship by applying modern statistical techniques. The current study was aimed to provide empirical bases for the presence of an ethnomedicinal culture in the Northern parts of Pakistan, where lies the great mountain system of Hindu Kush.

The beautiful Swat Valley which lies in the complex Hindu Kush mountain system possesses a surprising biodiversity due to its varied geographical and climatic conditions [6–8]. Swat Valley is known internationally for its green mountains and beautiful transparent fast-flowing rivers. The valley has varied topography with an altitudinal range of 700 to 6000 m above the average sea level (AASL), providing a complex biodiversity hotspot for researchers to be explored and protected. This complex topography gives rise to a complex biota and thus a surprisingly unique man-plant relationship (ethnobotany).

People of the area only living in the towns and cities have reasonable access to the modern health facilities, although a larger segment of the population living in the rugged mountains has little to no access to these facilities. Remote communities have established a connection to their immediate plant resources, which not only provide them with medicines but also act as an important resource of income generation, and are therefore of great significance to be understood, documented, and conserved [9–11].

Most of the inhabitants of the Swat Valley are the descendants of Akhozai subdivision of the Yusufzai Afghans (Pukhtuns/Pashtuns), living here for centuries [12]. The Kohistan area of the valley is primarily populated by Ghawris in the north while Torwalis resides in the southern rims. It is believed that the people of Indus Kohistan are Dardic, an old Indo-Aryan tribe [12]. Most of the residents of the valley are Muslims by religion with further subdivisions of Sayads (Saidan), Miaas (Miagaan), Sahibzadaas (Sahibzaadgan) and Mulaas (Mulayan) [13].

Swat Valley has mild to severe weather patterns influenced significantly by the altitudinal variation of the Valley. Phytogeographically, the area is considered part of the Sino-Japanese region with a monsoon season in the months of summer [6].

Considering the climatic variability along with the biodiversity and the land-use patterns in the area, eight agricultural and ecological regions can be recognised [6]. These zones are as follows: (1) The sub-tropical humid zone: this zone is predominantly the lowlands of the valley with characteristic short winters and extended summers. The indicator species are *Bauhinia variegata* (L.) Benth., *Phoenix spp.*, *Reptonia buxifolia* (Falc.) and *Nannorrhops ritchiana* (Griff.) Aitch.. (2) The sub-tropical dry zone: this zone covers most of the Swat Valley with the altitudinal range of 600 to 1000 m. The indicator species of this zone are *Acacia modesta* Wall. and *Olea ferruginea* Royle.. (3) The humid-temperate zone: the altitudinal range of this zone is from 1000 to 1500 m with hot and humid summer, especially, in June and July. The native tree species of this zone are *Pinus roxburghii* Sarg. and *Quercus incana* W. Bartram [6]. (4) The cool temperate zone: the altitudinal range of this zone is from 1500 to 2000 m with extended cold winter and petite summer. The common indicator species of the zone are *Pinus wallichiana* A. B. Jacks. and *Quercus dilatata* Royle. [6]. (5) The cold temperate zone: this is the most densely forested zone of the valley, ranging from 2000 to 2500 m altitudinally with *Abies pindrow* (Royle ex D. Don) and *Picea smithiana* (Wall.) Boiss. as the common indicator species [6]. (6) The subalpine zone: this zone is normally covered by snow for practically half of the year with an altitudinal range of 2500 to 3500 m. The indicator species of this zone are *Betula utilis* D. Don and *Quercus semecarpifolia Sm.* [6]. (7) The alpine zone and (8) the cold deserts are the highest points of the valley with an altitudinal range of 3500 to 6000 m above sea level. This zone lacks obvious macroflora.

It is obvious that these dissimilar ecological zones deliver micro-climates and habitats to an extensive variety of flora and fauna and are, therefore, endorsing to the establishment of complex man-plant ethnobotanical-cultures in the Swat Valley.

Ethnobotanical cultures can be protected if they are understood properly and the locals are given better stacks to hold on to. In many developed countries, especially North America and Western Europe, investors and the common public are equally, vigorously involved in the decision-making process at the managerial level and the opinions of the public are sought and considered before concluding on any future environmental policy [14, 15]. Common examples on the participation and consideration of public opinion can be seen in many cases in the UK, even on very minor issues, i.e. public consultation on hunting with dogs and other mammals [16, 17]. Scoring and quantifying the public opinion in environmental biology and ecology is sometimes an issue and requires large-scale ecological surveys and consultation of the public to make sure a reduced risk of human-wildlife conflict remains [10, 18, 19].

To extract and understand public opinion, questionnaires are considered as one of the cheapest and robust ways of obtaining large amount of quantitative data. Many examples include the usage of questionnaires in understanding the perception and attitudes of a community towards conservation strategies [7, 15]. Questionnaires are mostly aimed at the understanding of the actual behaviour of the subjects but also may focus on the hypothetical behaviour of the subjects [20]. The types and nature of the questionnaires can vary; for instance, closed-format questioning is a common practice in anthropology and ecology [15, 21]. Questionnaire surveys can be carried out in various means, i.e. post, telephone, and face to face interviews.

The review of literature of the ethnobotany of Khyber Pakhtunkhwa (KP) and Swat Valley shows that no one has ever attempted to quantify the use of plants or applied statistical indices to show their importance in the prevailing culture. In the oldest records, [22] studied the flora of the Upper Swat Valley while making key references to the ethnobotanical practices. Other regional study by [23], who conducted socio-phytological investigations on the Dabargai Hills of Swat District, identified some important plants of the area of therapeutic interest. Sulatanr Valley was investigated for its ethnobotany by [24] while [25] chose Malam Jaba mountains for the same. Cataloguing of important medicinal and economical plants was carried out by [26] of the Elum mountain. More recently, some scientists and armatures have expanded their work and studied large subvalleys of the Swat district [13, 27–32]. A few authors have attempted to specify their research on certain taxa and their ethno-ecological importance [33, 34], and some researchers [8] have attempted to explore the potential for in situ cultivation of MAPs and have obtained significantly positive results of the trials.

Most of the authors agree on the fact that the current practices regarding the use of MAPs and NTFPs are unsustainable and pose a serious threat to the biodiversity of the district. It is evident from the available literature on MAPs that none of the researchers have quantified the use of MAPs in Swat Valley. The current study is designed to understand the actual use of these plants through various quantitative measures like statistical indices, which could eventually help in understanding the versatility of some taxa and their links to the local ethnobotanical system. In the longer run, similar researches could help in developing fact-based strategies to conserve the flora and knowledge of the area.

Keeping in sight the prime objective of the study, questionnaires were designed to serve the precise purpose, i.e. to collect primary data from the inhabitants regarding the use of the plants, selection preference of the plants, and the conservation issues of the plants. The questionnaires included probe questions like as follows: how widespread is the ethnomedicinal knowledge in the study area? Precisely what plants constitute the cultural domain of the area? How aware are the people about the ecological relationship between trees and understory herbs? Is there any plant under serious stress from the anthropogenic stresses, i.e. exploitation by the traders? What stakes are there for the future biodiversity of the District Swat in terms of conservation?

## Methods

In the study, two questionnaire surveys were conducted to record the plant uses in a particular cultural domain and to document metadata about the peoples’ perception of the biodiversity and conservation measures in the area. The data was also used to calculate some very important statistical indices. The questionnaire prompts were designed to elicit information about specific quantifiable variables after [35]. This method was chosen because it is robust and inexpensive and is commonly used in socio-ecological surveys [15], but to the ethnobotanical survey in the area, constituting its initial application.

Questionnaire 1 mainly concentrated on eliciting background information about the ethno-medicinal knowledge flow in the community and ecological understandings of the locals along with some secondary information. Questionnaire 2 aimed at the recording of ethnomedicinal uses of plants in a particular ethnobotanical domain and was used in order to find out the intricate links between the social and physical environment of the area. Before the commencement of the study, permission was sought from the University of Reading Ethics Committee on the use of the questionnaires. The committee suggested some changes; permission was granted after the requests were fulfilled. A team of three qualified residents assisted with the field trips, in the understanding of local cultural norms and to avoid unnecessary antagonism with the inhabitants of the area. Respondents were randomly selected [15, 32, 36] to get a representative sample of the population and to get a less probable selection from the population. The Snowball sampling method [37] was used with some minor modifications. The main modification to the Snowball method was to select the most learned person of the community who can recall more uses of the MAPs. Therefore, the respondents were minimised as we achieved the representative samples of the population.

We also used semi-structured and free-listing questionnaires coupled with face-to-face interviews of which most of the population sampled was cooperative and willing to respond to. Residents were found to have considerable knowledge of the plant uses and were aware of the plants’ habitats. The elders had better knowledge of the recipes and were given priority for interviews. Interviews were complemented with forest walks and, in some instances, if the locals were willing, asked to show the plant in the wild. Data concerning local plants, i.e. their names, their uses, part-use, recipes preparations, and administration were recorded. In total, 116 participants were interviewed and had their completed questionnaires collected. Questionnaires were made available in three different languages, English, Pashto, and Urdu. Plant specimens were identified by experts in the Department of Botany of the Government Postgraduate Jahanzeb College, Swat and some by experts at the University of Reading, UK, after proper preservation. Some of the plant specimens were stored in the University of Reading’s Herbarium. A checklist of all the ethnomedicinal plants and their uses was developed (Table 1). Free-listing was carried out after [38] by asking the respondents to list all the plants used in the local cultural domain. Free-lists for 16 different diseases and health problems were collected. The recorded data for Questionnaire 1 was analysed by using Microsoft Excel and statistical analyses were done by calculating various indices (Table 2) from the data. The formulae used for calculating these indices are mentioned in the results section for better clarity.

**Table 1:** The 100 common medicinal and aromatic plants (MAPs) of the Swat Valley

No	Plant names	Part used	Family	Uses
1	*Justicia adhatoda* L.	Leaves	Acanthaceae	Used as an antispasmodic and bronchodilator
2	*Acorus calamus* L.	Underground Stem	Acoraceae	Used for its aroma in scents, known for its aphrodisiac activity and neurotoxicity
3	*Adiantum capillus-veneris* L.	Leaves	Adiantaceae	Scorpion bite, backache
4	*Viburnum foetens* (D.Don) Wall ex. DC.	Fruits	Adoxaceae	Fodder, fuel wood, fruits are eaten
5	*Allium sativum* L.	Whole plant	Amaryllidaceae	Curry ingredient, flavouring and aroma, used in blood pressure and is recommended in heart problems
6	*Mangifera indica* L.	Fruits	Anacardiaceae	Juice and shakes are common; refrigerant, digestive, laxative
7	*Foeniculum vulgare* Mill.	Whole plant	Apiaceae	Digestive disorders, flavouring agent, used in confectionery
8	*Senna alexendria* L.	Leaves	Apiaceae	Leaves are used as tea for treats ailments like cough and digestive disorders
9	*Cuminum cyminum* L.	Seeds	Apiaceae	Important part of Indian and Pakistani spices, wide use in different dishes Aromatic and carminative
10	*Coriandrum sativum* L.	Seeds	Apiaceae	Used in spices and known for carminative nature, flavouring agent
11	*Robinia pseudoacacia* L.	Whole plant	Apiaceae	Fuel wood mainly. Flowers are aromatic and attract bees
12	*Daucus carota* L.	Underground Root	Apiaceae	High vitamin A contents, common vegetable, and used as fodder
13	*Cassia fistula* L.	Fruits	Apiaceae	Fever, arthritis and neural disorders
14	*Caralluma edulis* (Edgew.) Hook.f.	Stem, bark	Apocynaceae	Very bitter plant, cooked and the water is drained, blood purifier, and good for skin diseases
15	*Periploca aphylla* Decn.	Leaves	Asclepiadaceae	Commonly used for feeding livestock, used as anti-fever
16	*Aloe vera* (L.) Burm. f.	Leaves	Asphodelaceae	Known for its effectiveness in skin problems, and kidney stone problems
17	*Artemisia maritima* L.	Leaves	Asteraceae	Anthelmintic and used as ethno-veterinary drug
18	*Cichorium intybus* L.	Leaves	Asteraceae	For gastrointestinal problems, for cuts and bruises, and for gall stones, etc.
19	*Onopordum acanthium* L.	Whole plant	Asteraceae	Ornamental and oil is also extracted from the seeds
20	*Berberis lycium* Royle	Roots, fruits	Berberidaceae	Stomach-ache, used in jaundice, and refrigerant
21	*Capsella bursapastoris* (L.) Medic.	Whole plant	Brassicaceae	Fodder and pot herb
22	*Nasturtium officinale* R.Br.	Whole plant	Brassicaceae	Pot herb and salad
23	*Brassica rapa* L.	Seeds	Brassicaceae	Pot herb, oil is used for cooking and is grown widely
24	*Buxus sempervirense* Hook. f.	Whole plant	Buxaceae	For wood carving and musical instruments
25	*Gymnosporia royleana* Wall. ex Lawson in Hook.f.	Leaves	Celastraceae	Honey bee plant, used in digestive problems
26	*Chenopodium album* L.	Whole plant	Chenopodiaceae	Pot herb and digestive
27	*Terminalia chebula* Retz.	Fruits	Combretaceae	Very potent in digestive disorders, and urinary problems
28	*Kalanchoe pinnata* (Lam.) Pers.	Leaves	Crassulaceae	It is used for its poisonous glycosides. It can act as a immune-suppresser
29	*Momordica charantia* Descourt.	Fruits	Cucurbitaceae	Used as vegetable, blood purifier, astringent and digestive
30	*Cucumis sativus* L.	Fruits	Cucurbitaceae	Eaten raw, low calories fruit (vegetable) common element of salad
31	*Citrullus lanatus* (Thunb.)	Leaves	Cucurbitaceae	Refrigerant, diuretic
32	*Cupressus torulosa* D.Don.	Seeds	Cupressaceae	For its oil, aroma, used for sore throat
33	*Diospyrus lotus* L.	Fruits	Ebenaceae	Fruit is eaten, leaves used as fodder, fuel wood
34	*Euphorbia helioscopia* L.	Whole plant	Euphorbiaceae	A poisonous plant, livestock suffer with severe health disorders if eaten
35	*Glycyrrhiza glabra* L.	Roots	Fabaceae	Mouth ulcers, peptic ulcers Expectorant and endocrinal suppresser
36	*Trigonella foenum-graecum* L.	Seeds, leaves	Fabaceae	Used as pot herb, for arthritis
37	*Arachis hypogaea* L.	Seeds	Fabaceae	Highly nutritious, eaten raw and roasted
38	*Vigna aconitifolia* (Jacq.) Marechal	Leaves	Fabaceae	Eaten as a vegetable and the ripe seeds are edible
39	*Quercus dilatata* Lindl. ex Royle	Whole plant	Fagaceae	Fuel wood, timber wood
40	*Quercus incana* Lindl. ex Royle	Whole plant	Fagaceae	Nuts are used as diuretic and the tree mainly as a fuel wood
41	*Fumaria indica* (Hausskn.)	Whole plant	Fumariaceae	A potent blood purifier, helps with body heat and used as tonic
42	*Gentiana kurroo* Royle	Leaves	Gentianaceae	Tonic, antispasmodic, febrifuge
43	*Pteridium aquilinum* (L.) Kuhn	Whole plant	Hypolepidaceae	Pot herb, cooked with yogurt and eaten with maize bread
44	*Juglans regia* L.	Stem, bark	Juglandaceae	Nuts are consumed as tonic; bark of the tree is used for gum infections
45	*Mentha longifolia* L.	Whole plant	Lamiaceae	Gastric and ulcers
46	*Ajuga bracteosa* Wall. ex Benth.	Whole plant	Lamiaceae	Sore throat, jaundice, astringent, and tonic
47	*Salvia moorcroftiana* Wall.ex Benth.	Leaves	Lamiaceae	Pot herb
48	*Thymus linearis* Benth.	Leaves	Lamiaceae	Coughs, cold, fever, rheumatism
49	*Mentha arvensis* L.	Whole plant	Lamiaceae	Very commonly known and used remedy for digestive problems and stomach-aches
50	*Mentha spicata* L.	Leaves	Lamiaceae	Used in different sauces, mixed with yogurt and salt
51	*Teucrium stocksianum* Boiss.	Leaves	Lamiaceae	Known for antispasmodic activity
52	*Micromeria biflora* (Buch-Hamp. ex D.Don) Benth.	Leaves	Lamiaceae	Known for its aromatic nature and its neural healing activity, also used as pot herb
53	*Vitex negundo* L.	Leaves	Lamiaceae	Antiseptic and antibacterial.
54	*Cinnamomum verum* J. Presl.	Stem, bark	Lauraceae	Aromatic bark is used in spices, especially in rice preparation
55	*Punica granatum* L.	Fruits	Lythraceae	Used in spices and for gastric problems. Juice is nutritious
56	*Malva neglecta* Wall.	Leaves	Malvaceae	Common herb cooked, digestive and emollient
57	*Melia azedarach* L.	Leaves, fruits	Meliaceae	Anthelmintic and vermifuge, also use for body heat release
58	*Morus nigra* L.	Stem	Moraceae	Digestive and laxative in properties
59	*Musa balbisiana Lacatan*	Fruits	Musaceae	Highly nutritious, tonic, juices and milk shakes
60	*Syzygium aromaticum* (L.) Merr. Perry	Fruits	Myrtaceae	Highly valuable plant; the fruits and flowers are used in curries
61	*Eucalyptus globulus* Labill.	Leaves	Myrtaceae	Used in teas, fuel wood, and bee plant
62	*Peganum harmala* L.	Leaves	Nitrariaceae	Another plant usually used for rituals like protection from evil eye, etc. famous for good aroma
63	*Mirabilis jalapa* L.	Leaves	Nyctaginaceae	food colouring, used as food, diuretic and purgative
64	*Olea ferruginea* Royle	Fruits	Oleaceae	The small fruits are eaten; leaves are used as antipyretic and antiseptic. Fuel wood
65	*Olea europaea* L.	Fruits	Oleaceae	Oil is used in cooking, massage and as anti-fungal, sacred plant
66	*Paeonia emodi* Wall. Ex Royle	Underground Roots	Paeoniaceae	Backache, tonic, epilepsy
67	*Papaver somniferum* L.	Latex, seeds	Papaveraceae	Known for neurotoxins and alkaloids. Seeds are used in confectionary
68	*Sesamum indicum* L.	Seeds	Pedaliaceae	Used in confectionery, high oil content
69	*Pinus wallichiana* A.B.Jackson	Whole plant	Pinaceae	Timber used in construction. Many other uses as well
70	*Picea smithiana* (Wall) Boiss.	Whole plant	Pinaceae	Timber tree, leaves used in teas, leaves used in mats
71	*Plantago lanceolata* L.	Whole plant	Plantaginaceae	Husk is used as laxative; the plant is anti-fungal in nature and is also cooked as pot
72	*Digitalis lanata* L.	Flowers, Leaves	Plantaginaceae	Cardiac stimulant, used in blood pressure
73	*Cymbopogon citratus* Springs.	Whole plant	Poaceae	Teas are prepared, sometimes mixed with green tea to enhance the flavour, diuretic
74	*Saccharum officinarum* L.	Leaves	Poaceae	Stimulant. Sugar crop in the country
75	*Polygonum biaristatum Aitch. & Hemsl.*	Leaves	Polygonaceae	Fodder, fish poison.
76	*Thalictrum falconeri* Lecoy.	Leaves	Ranunculaceae	Used in ophthalmic
77	*Aconitum violaceum* Jacq. ex Stapf	Underground stem	Ranunculaceae	Gout and rheumatism, aphrodisiac
78	*Ziziphus jujuba* Mill.	Fruits	Rhamnaceae	Fruits and leaves emollient, laxative and fruit is considered tonic
79	*Prunus amygdalus* Batsch.	Fruits	Rosaceae	Tonic, and used in neural disorders, served as a good gesture of hospitality
80	*Rosa indica* L.	Flowers	Rosaceae	Rose water is used for ophthalmic cures, digestive disorders, and scents
81	*Malus domestica* Borkh.	Fruits	Rosaceae	Well known tree in Swat, for its taste and aroma widely used, blood problems, nutritious
82	*Eriobotrya japonica* (Thunb.) Lindl.	Fruits	Rosaceae	Digestive, high sugar content, demulcent and expectorant
83	*Fragaria nubicloa* (Hook.f.) Lindl.	Fruits	Rosaceae	Fodder, fruit is eaten
84	*Skimmia laureola* (DC.) & Zucc. ex Walp.	Stem, leaves	Rutaceae	Highly salient plant in the community, used for the protection from evil eye, good aroma and used for digestive problems
85	*Citrus × limon* (L.) Burm.f.	Fruits	Rutaceae	Juice, flavouring agent in all sort of traditional dishes
86	*Aesculus indica* (Wall. ex Camb.) Hook.f.	Whole plant	Sapindaceae	Agricultural tools making fuel wood, fodder
87	*Dodonaea viscosa* (L.) Jacq.	Whole plant	Sapindaceae	Fuel wood, aromatic leaves are burnt in houses for some rituals
88	*Bergenia ciliata*(Haw.) Sternb.	Whole plant	Saxifragaceae	Wound healing, fractured bones
89	*Ailanthus altissima* (Mill.) Swingle.	Whole plant	Simaroubaceae	Fuel wood, considered as an alien species to the valley
90	*Allium cepa* L.	Whole plant	Solanaceae	Main ingredient of curry, spices and eaten raw
91	*Nicotiana tabacum* L.	Leaves	Solanaceae	Snuff tobacco is commonly used for making snuff
92	*Solanum miniatum* Benth.	Fruits	Solanaceae	Used for its laxative properties, some consider it as antispasmodic
93	*Capsicum annum* L.	Fruits	Solanaceae	Curry essential ingredient, a common spice
94	*Camellia sinensis* (L.) Kuntze.	Leaves	Theaceae	Diuretic and stimulant. Normally consumed after heavy meals
95	*Urtica dioca* L.	Leaves	Urticaceae	Used as pot herb anti-rheumatic
96	*Valeriana jatamansi* Jones	Whole plant	Valerianaceae	Antispasmodic and also used in cholera
97	*Curcuma longa* L.	Underground Stem	Zingiberaceae	Spices, cuts and bruises, carminative
98	*Zingiber officinale* Roscoe	Underground Stem	Zingiberaceae	Spice ingredient, used in curries, rice preparation, etc., refrigerant and carminative
99	*Elettaria cardamomum* (L.) Maton.	Whole plant	Zingiberaceae	A very well known for its aroma, a must ingredient of all spices
100	*Zingiber cassumunar* Roxb.	Underground Stem	Zingiberaceae	Used in beauty products and spices

**Table 2:** Abbreviation used for different indices calculated and their descriptions

No.	Index	Description
1	RFC	Relative Frequency of Citation
2	RII	Relative Importance Index
3	SI	Salience Index
4	PAR	Participant Agreement Ratio
5	CVI	Cultural Value Index
6	CII	Cultural Importance Index
7	CPI	Ali’s Conservation Priority Index

**Fig. 1:**
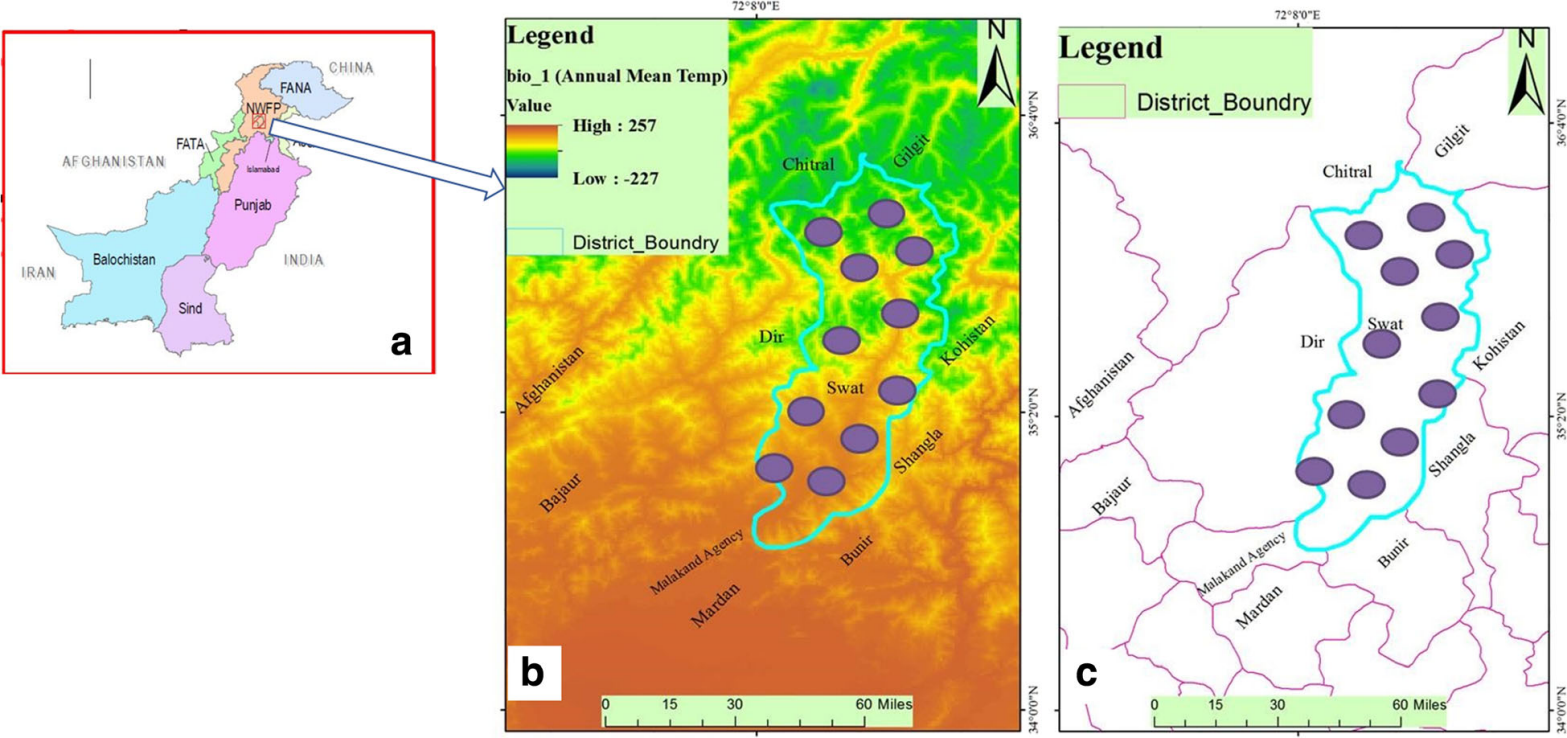
**a** The map of Pakistan and the provinces. **b** A thematic GIS map of annual mean temperature of the Swat Valley. **c** The administrative boundaries of the Valley and sampling sites

We have also used ArcGIS and WorldClim bioclimatic layers to develop thematic maps of the study area. The maps could provide visual interpretation of annual mean temperature layer in the study area correlating various areas of the valley with its high level of diversity (Fig. 1a, b).

## Results

**Fig. 2:**
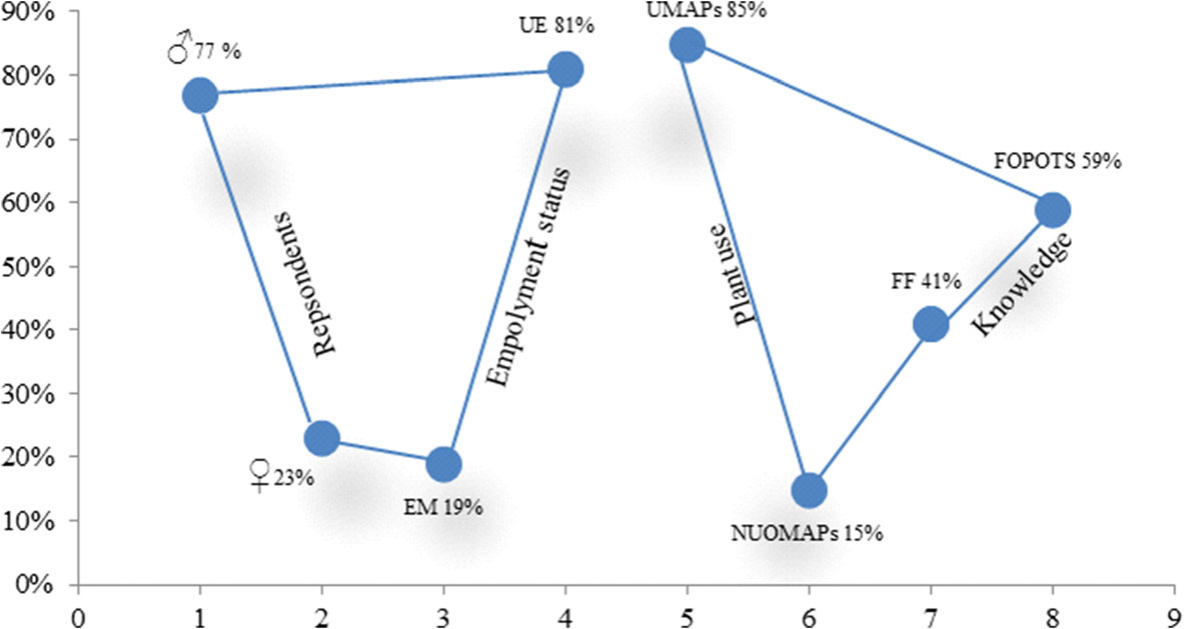
Respondent sex ratio and their employment status, use of medicinal plants (MAPs) and knowledge acquisition in Swat Valley are shown in percent. The percentage for different categories (♂ (male), ♀ (female), EM (employed), UE (unemployed), UMPAs (use of medicinal plants), NUOMAPs (no use of MAPs), FF (from family) and FOPOTS (from other parts of the society)) was calculated using excel and was superimposed into the graph configuration

The results obtained from questionnaire 1 indicate that the population sampled constituted 77% male and 23% female. It was also evident that only 19% of the population sampled was unemployed and 81% were employed, some of which are directly linked with farming and livestock husbandry (Fig. 2). It is clear from the results that 85% of people use medicinal plants for treating different ailments; only 15% did not use any medicinal plants directly for treatment of ailments, although the use of such plants was part of their daily food, e.g. used as pot herbs, in making curries. Over half of the respondents (59%) acquired the ethnomedicinal knowledge from different parts of the society, not restricted to learn it from their family hierarchical lines (41%) while 69% of the respondents had more than one family member with the knowledge of use of the same medicinal plant (Fig. 1).

**Fig. 3:**
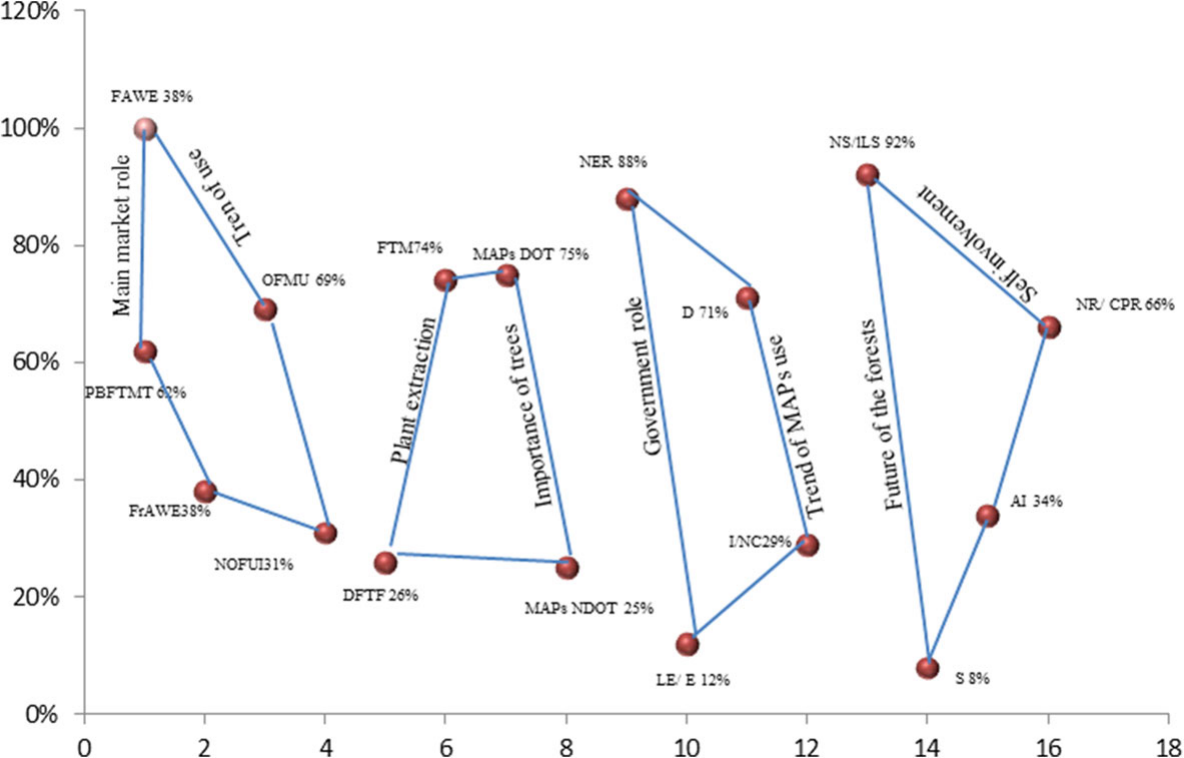
Shows the role of the main drug market, medicinal plant use in family, plant extraction, importance of the trees in the area, view about the government role, trend in the use of MAPs, future perception of forest conservation and self-role in conservation in Swat valley are depicted. The parameters, i.e. PBFTMT (plant bought from the main town), FAWE (from anywhere else), OFMU (other family member use), NOFUI (no other family use it), DFTF (direct from the forests), FTM (from the market), MAPsDOT (MAPs dependent on trees), MAPsNDOT (MAPs not dependent on trees), NER (no effective role), LE/E (less effective/effective), D (decreases), I/NC (increase/no change), NS/LS (not safe/less safe), S (safe), AI (active involvement) and NR/CPR (no role/cannot play role) are shown in percentage

Over 70% of the population were aware of the interactions between the presence of trees and the MAPs in association as a sub-flora (Fig. 3). Most of the respondents (88%) believed that the government were to blame for lack of response to the loss of the forest canopy and have claimed the current efforts towards the protection of the area’s forests as less-effective in comparison to the previous Swat State ruler. In the same manner, a great pessimism was found among the respondents as they consider that MAPs’ use is decreasing due to many reasons; the most noticeable being the gradual scarcity of the plants in the wild and high prices in the local market. Seventy-one percent of the respondents regarded a clear decline in the use of the ethnobotanical medicines. A majority (92%) of the respondents showed disappointment and pessimism towards the future of the forests in the area, while 66% of the respondents were either unwilling or unable to actively participate in the conservation measures (Fig. 2). This could be due to the limited available conservation projects which are predominantly run by non-governmental organizations (NGOs).

### Data analysis and results from questionnaire 2

The use reports (UR) for medicinal plants were calculated after [39, 40] on the bases of the ethnobotanical information: participant i states the usage of the plant species s in a use-category (disease/health issue) u. The terminology was adopted from [40] where NS represent species (s1, s2,……., sNS) in the entire number of use categories NC (u1, u2, ……uNC) while participants were denoted by N (i1, i2, …..iN). So, the highest value of URsui can be 1 when a combination is found or zero if no combination is observed; assuming the variables are constant the, URs can be expressed as:

1 $$ \mathrm{URs}=\overset{\mathrm{uNC}\kern1.25em \mathrm{i}\mathrm{N}}{\underset{\mathrm{U}=u1\kern1.25em \mathrm{i}=i1}{\kern1em \sum \kern2em \sum \kern0.5em }}\mathrm{UR}\ \mathrm{ui} $$

As indicated by the Eq. 1, the UR of all the respondents (from i1 to iN) are summed up in individual use group for the species (e.g. the number of respondents who specified the use of the plant species in the disease) and secondly all the UR of each category (from u1 to uNC) is summed, i.e. the entirety of all the uses of the species in the 16 diseases or health issues. Various indices were calculated (Table 2) to understand the cultural importance of the medicinal plant use and the hidden risk factors to their conservation.

### Relative frequency of citation (RFC)

This index is not dependent upon the variable of the use-category (see Eq. 2) and can be very simply extracted by dividing the frequency of citation (FC), by the numeral of participants (N) under the study [40]. FC denotes the number of participants recording the species, regardless of the use-category.

2 $$ \mathrm{RFCs}=\overset{\kern6em \mathrm{i}\mathrm{N}}{\mathrm{FCs}/\mathrm{N}=\kern1.75em \sum_{\kern1.75em \mathrm{i}=i1}}\mathrm{URi}/\mathrm{N} $$

### Relative Importance Index (RII)

This index was created by [40], based on the use categories of the species only and does not consider the sub-categories of the use [40]. The following equation (see Eq. 3) was used for calculating RII for 103 species used by the local community of the Swat District. In the equation, RFC (max) is the relative frequency of citation over the maximum while RNU (max) is a relative value of the use categories over the maximum (for more details see [40]). This index ranges from 0 to 1; 0 is where no one mentions the use of the species while 1 is where all the respondents indicate the use of the plants in all the use categories.

3 $$ \mathrm{RIIs}=\mathrm{RFCs}\left(\max \right)+\mathrm{RNUs}\left(\max \right)/2 $$

### Smith’s Salience Index (SI)

The free-listing interviews and questionnaires were very useful to calculate Smith’s Salience Index, which includes the frequency of mention and the spot of items in free lists [41]. The value ranges from 0 to 1, with 1 being highly salient. The average saliency is calculated for all the species across the different use categories. Using the symbology after [42], the following formula (see Eq. 4) was used, where rj is the place of the item j in the free-list, and *n* is the number of all the objects in the lists.

4 $$ \mathrm{Sj}=1\mathrm{rj}1/n1\ \mathrm{or}\ \mathrm{Sj}=n\mathrm{rj}/n1 $$

### Participant Agreement Ratio (PAR)

We used this to estimate the agreement of the community regarding the use of different plant species in each use category. Previously, this consensus analysis was called―informant consensus factor (ICF) by [5] but [43] named it as Respondents’ Agreement Ratio, while we are suggesting the term PAR. Using the formula (see Eq. 5) below, nur is the number of records in each class and nt is the quantity of plants used in that group. The range is from 0 to 1, where 1 shows the limited number of plant used in a use category, i.e. a high degree of consensus among the local population for the use of medicinal plants.

5 $$ \mathrm{PAR}=\mathrm{nur}-\mathrm{nt}/\mathrm{nur}-1 $$

### Cultural Value Index (CVI)

This index (CV in the original text of [44]) is one of the most useful indices used in ethno-biology, first introduced by [19] following the multiplication of the factors of [7]. The following formula (see Eq. 6) can be used for calculating the index, where three factors are multiplied together: NUs is the times of use categories, NC is the total figure of use groups, FCs is the number of participants mentioning the species to be useful in all categories and *N* is the total number of participants under study. The third factor is the sum of all the uses cited by the different participants in a category and is divided by the sum of the participants.

6 $$ \mathrm{CVs}=\left[\frac{\mathrm{NUs}}{\mathrm{NC}}\right]\times \left[\frac{\mathrm{FCs}}{N}\right]\times \left[\overset{\mathrm{uNC}\kern1.5em \mathrm{iN}}{\underset{U=u1\kern0.5em i=i1}{\sum \limits \kern1em \sum }}\right]\ \mathrm{URui}/N $$

For example, in the current survey, *Mentha longifolia* was reported to be used only in two categories, i.e. gastric problems and throat infections, so its NUs = 2; the total number of use categories are 16, so NC = 16 (NUs/NC = 2/16). The FCs value for *Mentha longifolia* is 27, as many people suggested the plant being useful out of 39 which is the total number of the respondents (N), e.g. (FCs/*N* = 27/39). Twenty-six people considered the plant being useful in gastric problems and one person recorded it being useful in throat infection, so the third factor of the formula would be 26/39 + 1/39. Combining all the three factors CVs or CVI = (2/16) × (27/39) × (26/39 + 1/39) = 0.0599. The range of this index is from 0 to NC, where 0 is the condition where there is no mention of the plant being useful.

### Cultural Importance Index (CII)

This is another useful index and is part of the cultural value index discussed above. As seen in the CVI, this particular index not only makes use of the quantity of use of the plant species but the spread of use in different use categories and was thus called by [40], the index of versatility of a species.

7 $$ \mathrm{CIIs}=\overset{\mathrm{uNC}\kern1.5em \mathrm{iN}}{\underset{U=u1\kern0.5em i=i1}{\sum \limits \kern1em \sum }}\mathrm{UR}\ \mathrm{ui}/N $$

The Cultural Importance Index is considered a sub-factor of the Cultural Value Index, and both have the same function, i.e. understanding the spread of a medicinal plant in a cultural domain (see Eq. 7).

### Ali’s Conservation Priority Index (CPI)

It is evident from the results above, that these indices can help explore the significance and versatility of plants in the field of anthropology and ethnobotany. However, they have no significance in estimating the conservation threat to this important flora. If we estimate the importance factor and calculate the plant part use in a particular culture by giving it a numerical value of a graduated scale in terms of their increasing risk, and then add it to the salience value and cultural importance index values of a plant, we can easily calculate a new index which we propose to be called as Ali’s Conservation Priority Index (CPI). The index can clearly demonstrate the extinction stress on a particular taxon. The index has proved to be very useful in identifying future vulnerable species in the study area and could be generalised to other places where no or little restriction persists from the authorities on the collection and exploitation of MAPs. Swat District is one of those regions where everyone has open access to the wild plant resources and can easily exploit the flora by over harvesting and over grazing, etc. The following formula (Eq. 8) for the index is recommended:

CPI = SI + RII + part-used value/3 (8)

In the formula, the SI is the Saliency Index of a plant species and the RII is the Relative Importance Index of the species under consideration. The Part-used value can be added from the Table 3 which signifies the importance in the distribution and extinction of a plant species. Those important MAPs which are used as a “whole organisms” in ethno-medicinal recipes or in any other economic or social practices of a culture are under highest risk of extinction and should be prioritised for conservation. The main condition of use of this index is that the plant under consideration must be the one collected from the wild. Plants of which only fruits or leaves are used in ethno-remedy or other socio-cultural practices are comparatively less prone to extinction. The value of the index ranges between 0 and 1, while 0 being no risk and 1 the highest risk and the one with the high value close to 1 should be prioritised for conservation in that region.

**Table 3:** Different plant parts and their quantitative use values in calculating Ali’s Conservation Priority Index (CPI)

S. No	Part used	Values
1	Whole plant and underground parts (roots, rhizomes, etc.)	1
2	Stem, twigs and latex (collected from injury)	0.75
3	Leaves, flowers	0.5
4	Fruits, seeds, natural excretes, i.e. gums, resins	0.25
5	Cultivated plants (for any plant part)	0.00

As trees can contribute many different parts which could be medicinally useful (i.e. wood, bark, twigs, leaves, fruits, seeds, resins), the value of the most important part is recommended for use. For example, if leaves and roots are both used as ethnomedicines, the value for roots shall be used from Table 3 for the calculation of CPI. The results suggest that over 60% of the recorded plants are herbs, 26% trees, 11% shrubs and less than 1% were climbers (Fig. 2). The angiosperm family of Lamiaceae was contributing nine species to the ethnomedicinal culture of the area, followed by the Apiaceae (7), Fabaceae (6), Rosaceae (5) and Solanaceae with (4) species. Asteraceae, Brassicaceae, Cucurbitaceae and Zingiberaceae contribute three while the rest of the families aid two species each.

The PAR values clearly indicate the highest degree of agreement. Over 8% of the residents agree on the choice of plants use for evil eye. This is followed by agreement on body cuts and psychological/neural disorders (about 8%). The highest PAR ranking for the use category of evil eye suggests that the people of the area still follow the old rituals and have a unanimous belief in the supernatural. Interestingly, the lowest PAR ranking was obtained for a very common health issue of headache (1.7%) (Table 4). Throat infections ranked fourth and gastric problems fifth in the ranking. Nutritional disorders ranked 15, suggesting that people have different approaches to maintain their dietary requirements and have low consensus on the plants consumed for solely nutritional purposes. This disagreement may also suggest that people are ill-informed about dietary requirements or a microculture exists in the different parts of the society.

**Table 4:** Participants’ Agreement Ratio (PAR) for 15 common diseases

No.	Disease/ailment	PAR	Agreement %	Rank
1	Evil eye	0.83	8.7	1
2	Body cuts	0.78	8.2	2
3	Mental disorders	0.76	8	3
4	Throat infections	0.74	7.7	4
5	Gastric problems	0.73	7.6	5
6	Tooth and mouth problems	0.73	7.6	6
7	Ear infections	0.68	7.1	7
8	Urinary disorders	0.64	6.7	8
9	Diabetes	0.63	6.6	9
10	Fever	0.6	6.3	10
11	Eye infections	0.53	5.6	11
12	Blood pressure	0.51	5.4	12
13	Skin problems	0.5	5.2	13
14	Body pain	0.41	4.3	14
15	Nutritional disorder	0.3	3.2	15

From the free-listing results, it is obvious that the centre of the Ethnomedicine of the Swat Valley, based on the values of Salience Index, consists of: *Berberis lycium*, *Thymus linearis*, *Mentha longifolia*, *Punica granatum*, *Curcuma longa*, *Ajuga bracteosa*, *Syzygium aromaticum* and *Skimmia laureola* (Table 5). The key plant of the domain is *Berberis lyceum* ranking highest in all five indices. *Skimmia laureola* is also one of the central plants of the ethnobotanical domain, ranking second in the SI, fifth in the RII, seventh in CVI and third in the Cultural Importance Index. It was in the 35th position in the CPI. *Mentha longifolia* ranked third in the SI and RII suggests that the plant is quite well known in the study area and is normally prioritised for its use. This plant also ranked high in the CVI and CII rankings, fifth and second, respectively. The fourth ranking of the plant in the CPI puts it in the list of high conservation priority species (Table 5). *Punica granatum* is ranked fourth in the Saliency and Relative Importance Indices. Its third ranking in the CVI shows its higher versatility in the local cultural. The plant ranked 72nd in the CPI, shows a very low risk of extinction and thus does not require any immediate prioritisation for conservation in the regional conservation policies (Table 5).

**Table 5:** The top 30 plants and comparison of their ranks based on their indices score

S. No	Plant Species	Ranks				
		SI	RII	CVI	CII	CPI
1	*Berberis lycium* Royle	1	1	1	1	1
2	*Skimmia laureola* (DC.) & Zucc. ex Walp.	2	5	7	3	35
3	*Mentha longifolia* L.	3	3	5	2	4
4	*Punica granatum* L.	4	4	3	5	72
5	*Curcuma longa* L.	5	6	4	6	3
6	*Ajuga bracteosa* Wall. exBenth.	6	2	2	4	2
7	*Syzygium aromaticum* (L.) Merr. & Perry	7	8	13	7	74
8	*Thymus linearis* Benth.	8	7	6	8	40
9	*Prunus amygdalus* Batsch.	9	10	10	10	73
10	*Mentha spicata* L.	10	11	15	9	102
11	*Allium sativum* L.	11	9	8	11	101
12	*Quercus dilatata* Lindl. ex Royle	12	16	21	15	5
13	*Micromeria biflora* (Buch-Hampex D. Don) Benth.	13	12	9	12	41
14	*Papaver somniferum* L.	14	13	11	14	75
15	*Nicotiana tabacum* L.	15	15	16	18	77
16	*Terminalia chebula* Retz.	16	14	12	13	76
17	*Brassica rapa* L.	17	19	19	17	78
18	*Thalictrum falconeri* Lecoy.	18	26	26	24	44
19	*Momordica charantia* Descourt.	19	23	24	21	79
20	*Gymnosporia royleana* Wall. ex Lawson in Hook.f.	20	17	17	16	42
21	*Eucalyptus globulus* Labill.	21	20	18	27	43
22	*Foeniculum vulgare* Mill.	22	22	23	20	7
23	*Mirabilis jalapa* L.	23	21	22	19	45
24	*Peganum harmala* L.	24	24	25	22	46
25	*Ziziphus jujuba* Mill.	25	27	28	25	80
26	*Fumaria indica* (Hausskn.)	26	18	14	23	6
27	*Pteridium aquilinum* (L.) Kuhn	27	25	20	26	8
28	*Aconitum violaceum* Jacq. ex Stapf	28	44	45	32	9
29	*Kalanchoe pinnata (*Lam.) Pers.	29	41	44	31	47
30	*Sesamum indicum* L.	30	30	31	34	82

*SI* Salience Index, *RII* Relative Importance Index, *CVI* Cultural Value Index, *CII* Cultural Importance Index, *CPI* Conservation Priority Index

Table 6 indicates that *Berberis lycium* Royle. has a high saliency value of (0.077) and shows the priority of use of the plant in the Swat District (Table 6). This plant also has the highest RII value (0.58) and the highest CVI (1.00). The highest value for CPI (0.6926) in Table 6 puts the plant in the highest threat level to extinction in the wild. *Skimmia laureola* was the second most salient (0.03) species of the area, and its familiarity and use makes it the second choice/option in the free lists, after *Berberis lycium*. The CVI value (0.02) puts the plant in seventh position in the ranking table (Tables 5 and 6). As the CVI values takes the use categories as well as the use reports into consideration, it is worth noting that *Curcuma longa* is not a plant locally grown or collected from the wild, but is imported from other parts of Pakistan or India. This pattern shows strong trade links of the local markets with national and international markets, and there is clear potential for growth in both imports and exports of the important MAPs. This may also mean that plants like *Curcuma longa* should be tried for cultivation in the valley, so that the import revenue is saved for other growth sectors. Cultivated plants have a great potential in the area, e.g. *Allium sativum* and *Allium cepa*, are not just widely cultivated and used in spices, i.e. curries, but are also well known for their therapeutic properties.

**Table 6:** The top 15 plants and comparison of their indices scores

Plant Names	SI	RII	CVI	CII	CPI
*Berberis lycium* Royle	0.077	0.58	1	1.33	0.69
*Skimmia laureola* (DC.) & Zucc. ex Walp.	0.038	0.25	0.02	0.66	0.27
*Mentha longifolia* L.	0.036	0.27	0.05	0.69	0.36
*Punica granatum* L.	0.032	0.27	0.13	0.58	0.13
*Curcuma longa* L.	0.029	0.25	0.09	0.53	0.37
*Ajuga bracteosa* Wall. exBenth.	0.028	0.31	0.21	0.61	0.41
*Syzygium aromaticum* (L.) Merr. & Perry	0.023	0.16	0.01	0.41	0.09
*Thymus linearis* Benth.	0.019	0.17	0.03	0.35	0.18
*Prunus amygdalus* Batsch.	0.018	0.14	0.01	0.3	0.09
*Mentha spicata* L.	0.017	0.14	0.008	0.35	0.008
*Allium sativum* L.	0.016	0.15	0.02	0.3	0.013
*Quercus dilatata* Lindl. ex Royle	0.016	0.1	0.004	0.25	0.34
*Micromeria biflora* (Buch-Hampex D.Don) Benth.	0.014	0.14	0.01	0.28	0.17
*Papaver somniferum* L.	0.012	0.13	0.01	0.25	0.09
*Nicotiana tabacum* L.	0.012	0.1	0.007	0.2	0.08

*SI* Salience Index, *RII* Relative Importance Index, *CVI* Cultural Value Index, *CII* Cultural Importance Index, *CPI* Conservation Priority Index

## Discussion

The herbal market of the major city (Mingora) of the valley plays a significant role in the continuous supply of MAPs to the major part of the community (62%). This may be due to the active role of the medicinal plant traders who normally hire local people as cheap labour for harvesting these plants from the wild and directly supply them to the market. However, this also shows the potential of further expansion of the MAPs trade, if an eco-friendly and sustainable approach is adopted.

Part of the study followed the basic pattern of cataloguing of plants and their uses like [22–24, 28, 30]. People perception has confirmed the findings [45] that the MAPs are under severe stress from overcultivation and deforestation. The need for conservation is ever high for these important plants [29].

People in most of the subvalleys of the Swat district have similar uses of these plants, as [29] confirms that from the subvalley of Shawar and Madayan. The results also confirm the findings of [32] which confirms the complex structure of the ethnobotanicals’ market. We can also confirm results that most of these plants share similar use categories in Swat districts. The results of [34] are in corroboration with our results which suggest that the overall population density of each plant is decreasing.

Domestication and ex situ conservation of the some therapeutically important medicinal plants have greater economic potentials [8]. Their tests on the domestication and cultivation of six medicinal plants in farm lands have positive results [8]. This confirms our observation that the plants imported from other regions could be tested for cultivation which could help boosting local economy.

Almost all the investigators agree on the fact that Swat is potentially a biological hot spot, not just for the country, but for the whole of South Asia, and can act as a trade hub of MAPs and Non-Timber Forest Products (NTFPs) [8, 13, 30, 32, 34]. Most of these authors agree that the current practices, regarding MAPs and NTFPs are unsustainable and pose a serious threat to the biodiversity of the district. Threats like deforestation and uprooting of medicinal plants [29] provide sound bases for the use of Ali’s Conservation Priority Index to quantitatively measure the threat level of individual plant species. Some researchers [30, 34] reported the habitat fragmentation and unwise use of these plants are the severe threats to their extinction. This means that when these plants lose their habitats they will lose their use in the traditional recipes and will eventually be excluded from the ethnobotanical domain of the area which could lead to more stress on the already ineffective government health facilities.

The current study is of great significance to the public health policies as it highlights the importance of the locally available natural phyto-products used by the residence. The greater majority of the ailments treated with the locally collected MAPs are reducing the stress on the scarcely available public health facilities of the area and at the same time saving financial resources of the destitute residents.

## Conclusions

District Swat possesses remarkable biodiversity owing to its varied topographical and climatic conditions. The use of medicinal and aromatic plants in the valley is a common practice, as 85% residents used botanicals to treat various ailments. The common knowledge of use of the important flora in the area makes a unique and well-established ethnobotanical culture. The use of these plants shows a decreasing trend which can be clearly attributed to factors such as uncontrolled harvesting, insignificant role of the government towards conservation and presumably the lack of conservation knowledge and strategies; these factors will soon irreversibly damage the floristic biodiversity of the area, and consequently, the rich ethnomedicinal knowledge will vanish forever. It can be established, by interviewing the locals, that most of residents are well aware that the on-going degradation of the forest canopy is severely harming the sub-flora; however, most of them are unwilling or unable to effect change. There is also a blame game being played in the valley; locals are blaming the government and vice versa. The study has established that people and plants have a deep intricate relationship, and the people of the area have developed a certain cultural domain of the plant use. This also confirms that not all plants of the valley are equally utilised as herbal remedies but rather some plants are more versatile than others and have significantly more impact on the prevailing ethnobotanical culture.

The research has tried to establish a new concept of threat level to plants and highlighted index for conservation priority. This new index can be applied to areas where plants are openly collected from the wild. The index can calculate the extinction risk to a taxon, using the plant part used as an important factor. Plant parts used are given categorical values (Table 3). This index can also be defined as the extinction risk factor. The calculation of the index requires the Smith’s salience index value [41], as this value is the primary use of a certain item by persons or population. Adding the Smith’s Salience and Cultural Importance Indices which are the measures of versatility-diversity of use [18, 40], together with the part use value, and dividing them by three get the average value. The maximum theoretical value for CPI is 1, suggesting the plant needs to be put in the high priority list of conservation. If the plant is well known and highly versatile and, if its part use is putting it in danger of extinction, the stakes for its conservation would be very high.

The prime objective of the study was to understand the cultural domain of the ethnobotanicals use of the valley. The objective was achieved by concluding that only a few important plant species make the cultural domain of the local community. It was the first ever attempt to establish this fact systematically in the area. The usual trend of ethnomedicinal researches from the area focuses on the compilation of plant uses in general. This study is unique as it has quantitatively evaluated the ethnomedicinal knowledge in Swat Valley. We highly recommended that studies of similar nature in the area be conducted on a much wider scale, to document and understand the socio-cultural practices of the area and to envisage a policy for conservation of the flora and the invaluable knowledge related to it.
